# Creative forms: booklets by the hospital senses collective

**DOI:** 10.1136/medhum-2023-012638

**Published:** 2023-06-28

**Authors:** Marie Allitt, Agnes Arnold-Forster, Harriet Barratt, Victoria Bates, Rebecka Fleetwood-Smith, Clare Hickman

**Affiliations:** 1 University of Edinburgh, Edinburgh, UK; 2 York St John University, York, UK; 3 University of Bristol, Bristol, UK; 4 Newcastle University, Newcastle upon Tyne, UK

**Keywords:** Built environment, Medical humanities, Literature

## Abstract

This article details the creation of a series of booklets designed to explore sensory encounters with hospitals and healthcare environments. The booklets were devised as a series of prompts or provocations, created to attend to and examine embodied, sensory encounters with health/care settings rather than to present research findings. Bringing together an expansive range of backgrounds and skill sets the booklets were created to sit within and beyond language through their design, form and content. Within this article we share the ways in which the works are deliberately unfinished and exploratory as this necessitates that those interacting with them create their own meanings and explore how they think and feel about health/care environments. The form and design promote a certain attentiveness and embodied engagement. For example, users must engage with the works carefully, gently turning and unfurling the fragile pages. This is further illustrated through qualitative insights collected from users of the booklets. Throughout this paper we argue for multiplicity in the ways in which we explore and present sensory-focused research. Our attention to multiplicity is supported not only through the design, form and content of the physical booklets but through the creative audio description, text and images created to complement and support these works. These are available online to ensure that our provocations are widely accessible. Within this paper we critique how a reliance on narrative form can limit the ways in which we engage with spatial, sensory and emotional concepts. Such concepts are by their very nature challenging to articulate and arguably require more-than-text-based approaches. We propose that embracing creative, exploratory and seemingly risky routes to examining and presenting such concepts is critical in expanding research.

## Introduction

A familiar scene: a group of researchers, towards the end of planning a research project, wish to disseminate their thinking in a creative and accessible way. The impulse might be to use a visually engaging summary report or an exhibition of work. There is certainly value in such creative dissemination of research projects, as part of a commitment to accessible public engagement. In this article, though, we argue that sensory, creative research forms are also a methodology and a practice in themselves; they are not only an ‘added extra’. They can be an alternative form of academic and critical engagement, rather than only outreach or dissemination. Our topic, the sensory environment of hospitals and other settings of healthcare and care, benefits from using creativity to explore and think through the multiple ways that people experience, understand and represent its phenomena.

We argue that, for researchers of the senses and the medical humanities, it is limiting to start with a traditional academic publication which does not necessarily do justice either to the subject matter or to collaborative and interdisciplinary ways of working. Instead we consider how the ‘provocation’ approach and the physical form of research outputs, such as the multisensory, cross-practice booklets we discuss here, are themselves a key enabler of critical thinking. As Matt Hayler argues, ‘[f]orm and content are always entangled, with forms supporting, competing with, or codetermining the meanings of their contents’ (Hayler 2021, 83). This argument builds on and supports a range of scholarship on the ways that books and booklets—as objects, not just as vessels for information—can help people to think critically, and to use embodied ways of reading (and, in some places, listening, smelling, touching) to enhance their thinking ([Bibr R14]; Bolaki 2020; Lupton and Lipps 2018). It builds on work in the medical humanities that explores the value of form and genre for exploring themes around health and the senses, including artists’ books (Bolaki and Čiricaitė 2017), comics (Williams and Annandale 2020), zines (Cooper 2023) and multisensory digital magazines (Stehlikova 2020-). There is a growing body of literature advocating for the value of such forms as facilitators of critical thought and embodied knowledge. However, their full potential remains largely unrealised; many researchers continue to view such creative outputs as ‘nice to have’ or public engagement strategies rather than integral parts of research. We seek to show the value for medical humanities researchers of embracing alternative material and multisensory forms, as part of knowledge production.

To do so, this article presents the work of the ‘Hospital Senses Collective’, which in 2020–2022 created a collection of multisensory booklets in collaboration with a range of other multidisciplinary academics, healthcare workers and creative practitioners. Our booklet series includes short coauthored provocations, creative commissions and a playful physical form that help us to explore research questions in ways not possible in traditional publication formats. Through these varied approaches the booklets invite the user to contemplate the diverse sensory environments of six hospital spaces—Wards, Corridors, Waiting Spaces, Thresholds, Operating Theatres and Laundries. They are designed to be an open, unfinished, relational dialogue between the booklets and the person using them. At the core of our collaboration is not a shared research project, but an ongoing conversation about interdisciplinarity and new ways of doing research, both within and beyond a core group. A different type of publication is necessary, as a way of exploring opportunities, asking questions and promoting dialogue. We use the term ‘users’ as opposed to ‘readers’ when exploring how people encounter the booklets; this is an attempt to reflect the multisensory ways in which people interact with the booklets, whereby meaning is derived from the form and content as well as text.

### Senses and the hospital

Sensory engagement is particularly important for those researching, working in, designing and experiencing healthcare environments which are emotionally and sensorially sensitive (McCurdy 2018). There are some works on senses in hospital spaces, in relation to historical sites (Bates 2019; Bates 2021; Reinarz 2012; Theodore 2018) and contemporary ones (Duque et al. 2019; Pink et al. 2022; Stenslund 2015; Sumartojo et al. 2020). There are also some evocative descriptions of the sensory experience of hospitals in some patients’ memoirs (e.g. Mantel 2010 and Gleeson 2019). Other institutions like schools (Grosvenor 2012; Landahl 2011; Lawn and Grosvenor 2005) and prisons (Herrity, Schmidt, and Warr 2021) have also been the subjects of a rich and flourishing scholarly literature. However, the hospital is unique. It is a place where the body is subjected to manipulation and intervention; scrutinised under microscopes, in MRI scanners, via X-rays; and a space of emotional intensity where life, loss, love, frustration, hope and disappointment are acutely felt.

As our project makes clear, hospitals are not just buildings with floor plans or structures with exterior walls. They are inhabited, and thus constituted, by people. Patients, staff and visitors all make meaning out of hospitals through design, decoration, the bringing in of objects and adaptation. Indeed, as Sherry Turkle argues, ‘we are on less familiar ground when we consider objects as companions to our emotional lives or as provocations to thought’ (Turkle 2007, 5). Materiality, objects and the senses have traditionally been marginalised in accounts of healthcare precisely because they are seen as irrelevant, or even obstructive, to considerations of thought, intellect and sense-making. As Turkle continues, ‘behind the reticence to examine objects as centrepieces of thought was the value placed, at least within the Western tradition, on formal, propositional ways of knowing’ (Turkle 2007, 6). Studying place, space, inhabitation, materiality and the sensory is a crucial part of understanding what hospitals mean and how they are experienced and felt. To do so via creative methods further expands the potential for meaning-making within alternative modes of knowing.

People engage in ‘place-making’ practices, and the senses allow us to reconsider the related but distinct concepts of ‘social space’ and ‘lived-in place’. It is also crucial to recognise and explore the complex temporality of the senses in healthcare and care settings. Scholars need to treat spaces as heterogeneous and relational rather than merely snapshots of moments in time (Jameson 1991; Lefebvre 1991; Massey 1994). The sensory environment of hospital spaces does not just change over time with shifts to design and inhabitation; there are a range of space-time relationships and forms of atmospheric production that occur within any given space *at* a specific point in time. Our booklets show how sensory experiences of hospital interiors and objects populate the designed ‘space’ of the modern hospital with meaning and matter, thereby turning them into ‘places’.

### Making the booklets

A traditional academic output is not always the best form for critical thinking in relation to sensory experience. In planning our booklets, we sought to explore how a more creative and multisensory form might enhance critical thinking about hospital senses. We see these works as complementing academic works in the medical humanities and sensory studies; they are also tools for critical thinking and are not simply ‘nice things to look at’. We designed these booklets to sit within and beyond language, and to be deliberately unfinished and engaging. There is great value in offering questions and provocations in a range of creative forms, with these prompts grounded in sensory research and knowledge, but without giving neat answers or telling people how to feel and think. As noted below, in the section on ‘Encountering the Booklets’, people bring their own experiences and feelings to the booklets and co-produce meaning. This is particularly true when considering a place such as the hospital which will have been encountered by most users of the booklets for one reason or another. We have, therefore, left space for users to consider their own sensory memories and experiences in relation to the booklets rather than filling in all the gaps for them.

The individual sections of each booklet are formed around a prompt or provocation: a literary quote; a photograph; an object; a feeling; a creative piece. In places, we analyse and discuss these prompts, to offer our own reflections or to ask questions of the user. In others, we leave the prompts to speak for themselves, in order to create an active dialogue or relationship between the user and the booklets. In creating these booklets, we wanted to recognise that hospital spaces are not static: as Laurinda Abreu and Sally Sheard demonstrate, ‘Hospitals are *living* entities, they are more than the individual components of buildings, staff and patients’ (Abreu and Sheard 2013, 1). They are palpably felt, breathed, seen and lived by those who encounter them. We see these booklets as another form of encounter, and they are designed to acknowledge the impossibility of giving a comprehensive account of ‘the’ sensory environment of hospitals. These prompts and provocations mean that each individual user is invited to think about the sensory environment of the hospital in a way that is meaningful to them. We did not design the booklets as a research ‘output’ in the sense of a finished set of ideas, but as an invitation for people to consider the hospital and its many spaces in more multisensory ways.

The booklets move through the different spaces of the hospital to illuminate the multisensory experience. We identified six spaces for the booklets: Waiting Spaces, Thresholds, Wards, Operating Theatre, Laundry and Corridors. We organised the booklets spatially in order to encourage the user to think in terms of moving through the hospital and its different environments. Some of the booklets cover specific types of space (laundries; corridors; wards; operating theatres). Others cover experiences associated with a range of spaces (waiting). We also included conceptual spaces (thresholds) to encourage an engagement with intangible as well as tangible hospital spaces. This structure in itself highlights the tensions of following a traditional hospital plan and opens up places for dialogue between concrete spatial elements such as the waiting room and more uncanny sensory experiences such as moving from one state to another (from onlooker to patient; from waking to anaesthesia). This dialogue between the visible and invisible is reflected in the use of both traditional written forms and more creative responses which allow us to encourage a depth and richness of engagement from readers. These booklets have captured just some of the multitude of hospital experiences. While we have striven to include a range of spaces, there are undoubtedly other areas that could have been included. There are also more perspectives that could be drawn on, especially the forgotten or marginalised people who spend time in hospitals and other healthcare and care settings. We have tried to work towards this by including some of the voices typically overlooked, such as a porter (see Crane 2022) and the perspective of an older person, but there inevitably remain gaps.

As a team, or ‘hospital senses collective’, we coauthored a number of pieces responding to prompts and offering provocations. These include historical details, illuminating how different hospital spaces have their roots in, for example, nineteenth-century asylum architecture, or the 1940s with the birth of the National Health Service, as well as insights into how certain spaces have evolved and developed. They also feature contemporary details of hospital spaces, such as art installations, co-designed spaces and textiles. These written pieces bring together the multidisciplinary expertise of the collective, prompting people to consider the value of thinking about the sensory environment of the hospital through architecture, culture, history, objects, psychoanalysis and psychology. We also explore the hospital from different perspectives. How does the waiting space sound to the person awaiting news of their husband’s procedure? What does the infamous hospital food taste like? How does it feel to walk the hospital corridors all day, transporting patients and resources? What does the bed linen smell of? What does it mean to cross from the hospital foyer into the garden, or car park, or smoking area? What does the hospital corridor ceiling look like for the patient moving wards? We do not see this list as exhaustive in terms of how the hospital might be experienced, but instead offer examples and snapshots to provoke users to reflect on their own experiences of different spaces, as this extract from our ‘Routes In’ to Thresholds demonstrates:

Thresholds are points of crossing. In hospitals there are many different crossing points, whether between physical spaces, states of being, or knowledge. The most obvious is the one from the world outside: from car parks and trees, into the inner spaces defined as the hospital. Walking or being wheeled across that line changes your status: for some, this line makes them a patient, subject to the authority, knowledge, and expertise of the hospital system. For others, crossing the line marks a different threshold: from off duty to on, from ‘general public’ to staff member. On crossing this line, every person meets the unmistakable sounds, sights, and smells of the hospital. (Hospital Senses Collective 2022)

**Figure 1:**
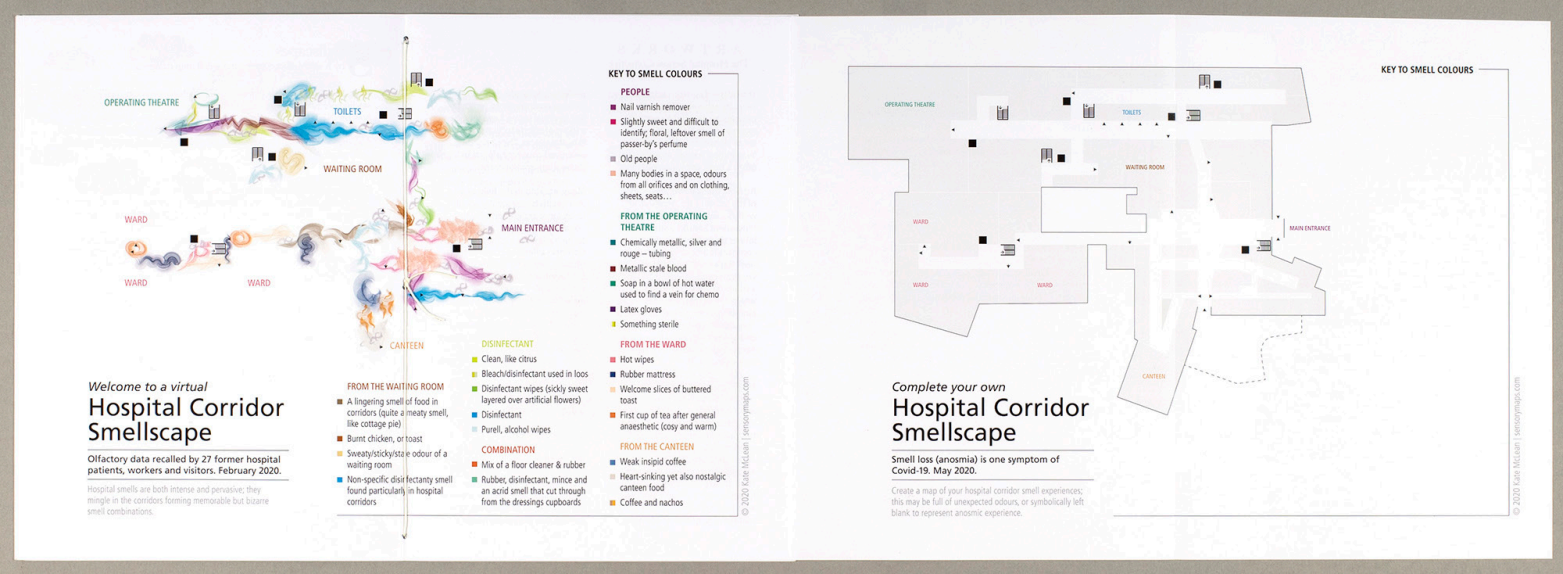
Kate McLean, ‘Hospital Corridor Smellscape’, https://hospitalsenses.co.uk/smellscapes/. Republished under CC BY-NC.

The pieces are deliberately wide-ranging and disparate, intended as openings to a dialogue with an interested user who may gravitate towards certain questions, prompts or provocations. Woven throughout each booklet are commissioned works. Some of these offer alternative views of the hospital from perspectives that are often neglected, such as a written text by a hospital porter. Many others are creative commissions which are deliberately wide-ranging, including poetry, visual artwork, and forms of intermedia such as photographic representations of sculpture and an artistic interpretation of a musical score. These creative pieces are also prompts and provocations, sometimes offered with context or interpretation, and in other places sitting simply in an open space waiting for dialogue with the user. Some of the pieces are designed to be annotated or engaged with, such as a blank outline of a hospital plan that sits alongside the hospital corridor smell map (figure 1). Other creative contributions to the booklets provide visual interpretations through artworks, which enhance and speak to the text rather than simply ‘illustrating’ it. These creative pieces highlight and engage with the limits of prose language for exploring sensory experience, and we view them very much as—like the prose pieces—a starting point for thought, rather than simply creative representations of different sensory experiences of the hospital.

Our aim has been to offer a collection of responses to the sensory and spatial experiences of the hospital. The voices, skills and insights from artists, musicians, hospital staff, clinicians, designers, poets, come together to offer some multisensory, multispatial insight into the diversity of hospital experiences. The commissions create moments of reflection, exploring varied hospital senses through a rich array of lenses and approaches. Within academia, creative research methods are often positioned as facilitative of a process of defamiliarisation whereby, through a process of creative engagement, we may rethink and reconsider the familiar (e.g. Mannay 2015). We have similarly adopted this process of defamiliarisation by placing creative commissions and academic contributions alongside one another, inviting us to rethink and reimagine past, present and future hospital environments through the senses.

As discussed below, we have also included material sensory prompts in our booklets, such as pieces of a surgical gown or a piece of soap. Some of these are deliberately unfinished, such as a playlist of surgical music to which users can add their own choices. By offering some of these prompts without analysis or interpretation, we also seek to account for the presence and agency of material objects, virtual technologies, animals, plants, temporalities, and other non-human or more-than-human entities in hospitals. We give these non-human entities their own space to exist outside of our linguistic descriptions or representations. This approach supports the work of scholars of science/technology studies, space and place who have also explored relations between different ‘human’ and ‘non-human’ actors, relations which have been variously explored in terms of ‘non-representational’ approaches, ‘actor-networks’ and ‘assemblages’ (e.g. see Deleuze and Guattari 1987/1980; Ingold 2000; Ingold 2011; Latour 2005; Massey 2005; Thrift 2008). These approaches emphasise relationality, interactions and processes. The design of our booklets similarly seeks to explore how healthcare environments have been made or co-produced, rather than built or designed, over time.

It is also important to note that we do not see our written pieces as separate from, or significantly different to, the multisensory and artistic prompts. While we have described artistic pieces above as ‘creative commissions’, we also designed our written pieces to be creative. The text does not act in opposition to the creative content, but also provokes and offers a multidimensionality. We have shaped the text to become an object, to act as a space. This shaped style, mimicking the corridors, the gapping of waiting spaces, becomes ‘a form of verbal architecture’ (Bellolo 1970, 41). The text speaks as much as it shows, and encourages us to embody the space. We have used the ‘Routes In’ of each booklet to bring the content and form together in embodied and shaped experiences. Can one experience the sensation of waiting, for example, by following text that is stretched and spaced out?

Similarly, the interactive, multisensory and creative pieces in our booklets are not in opposition to language and critical thought. Such creative pieces are a fundamental part of exploring critical and complex questions. They offer routes through the limits of language, and ways to think about embodied forms of knowledge. They also offer spaces and opportunities for active reflection on the themes raised in written pieces. Our theme, the senses, lends itself particularly well to exploring these forms of embodied knowledge and practices. Our intention is to show that more diverse ways of knowing, exploring and thinking should—and can—be a core part of mainstream academic work in the medical humanities and critical thinking, rather than being treated merely as routes to impact or outreach (see also Allitt et al. 2022).

### Using the booklets

The material qualities of the booklets, their design and form have been carefully considered to promote specific interactions and engagement. Each booklet embodies themes concerning hospital senses, allowing us to move beyond the visual to explore the spatial, temporal and sensorial through design and form. For example, users meander down the ‘Routes In’ of Corridors; they reveal missing text through the turning of tracing paper; their gaze is directed through viewfinders; they unfurl maps and are invited to contribute their own hospital smellscapes. The narratives are non-linear: they require ‘reader interactivity, [and] introduce structural and semantic complexity’ (Whittaker 2021, 233). These processes and interactions ask the user to spend time with and in each booklet, allowing them to explore and challenge understandings concerning the hospital environment.

The forms invite, even necessitate, that those engaging with the booklets use their bodies: engagement with the booklets is, therefore, an embodied practice. As Lupton and Lipps put it, ‘sensory design confronts the body’ and ‘objects gain meaning and value in our embodied experience of them’ (Lupton and Lipps 2018, 33). The form and vulnerability of the booklets—they are printed but hand sewn, with delicate and fragile parts such as fabric and tracing paper—requires physical intimacy and care. Users must be gentle and move slowly through the booklets. As Randi Annie Strand notes in relation to her book *Arabesque 3*: ‘quick movement is physically impossible’ and ‘fragility and vulnerability appeal to care and consideration’ (Strand 2017, 90).

**Figure 2:**
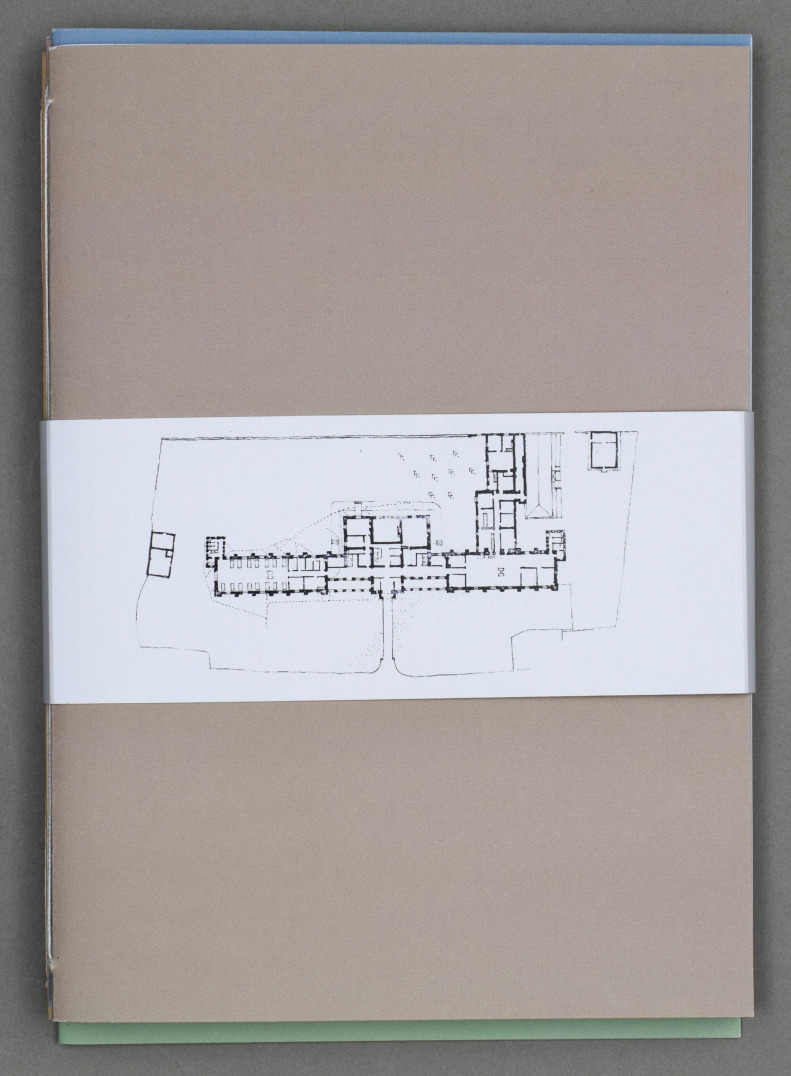
Image of belly band. Republished under CC BY-NC.

By encouraging users to reflect on the embodied experience of the hospital, we also invite them to engage with these booklets through physical and sensory experiences. The hospital floor plan printed on the belly band (figure 2), which wraps around and keeps each set of the booklets together, sets a tone of interaction and enquiry, echoing the sentiment of the book project *These Pages Fall Like Ash* (Abba et al. 2013), which declares: ‘The book is a map // everything in it is a path to be walked’. Elsewhere, the shape of our text speaks to the subject matter and calls for particular types of embodied reading practice. The surgical viewing gallery, for example, mimics the angles of the operating theatre, with sections on a slant or upside down. The ‘Routes In’ of ‘Waiting Spaces’ offers blocks of separated text, which stops and slows the user, and forces your body to bridge the gaps. You slow down. You pause, using the text as stepping-stones to enter the healthcare spaces. This shaping of the text renews attention to the body, encouraging the user to reflect on how their body is forced to move and act in certain spaces. This contributes to the physicality of the reading process. The shaping of the text does not seek to limit or bound users’ experience of space. Where the hospital intentionally inhibits us from straying from the path, preventing desire lines, these representational forms allow the user to inhabit the hospital spaces in different ways.

**Figure 3:**
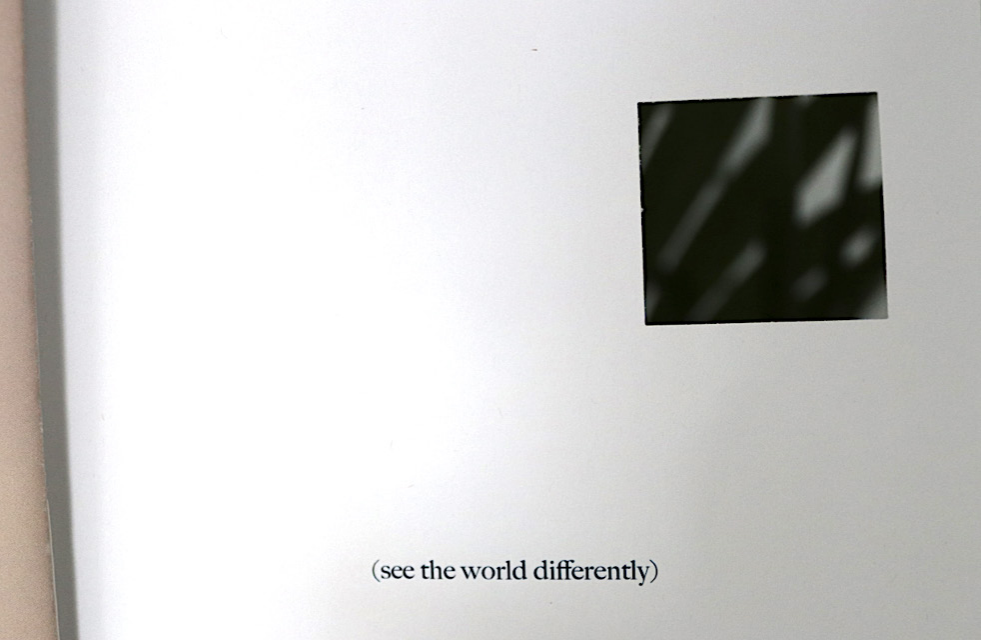
Viewfinder. Republished under CC BY-NC.

In places, the booklets encourage people to look away from them and to engage with the world around them in new ways. In ‘Thresholds’, for example, we were inspired by the idea of looking out of the hospital window and offered a ‘viewfinder’ through which users could—to quote the booklet—‘see the world differently’ (figure 3). This viewfinder invites an embodied and attentive form of close, slow viewing; it is not a rejection of the visual in favour of the ‘sensory’ but, instead, an invitation to the user to think closely about the act of seeing and how they engage with space and place. As Fiona Johnstone remarks, the visual is ‘an embodied perceptual experience that also involves the other senses, and which often has an affective quality’ (Johnstone 2018). The viewfinder also encourages people to move around with the book, which offers another form of embodied reading that engages movement and the sense of proprioception. As Jonathan Dovey, Tom Abba, and Kate Pullinger argue in their work on ambient literature, ‘the act of walking and attending to both the content and the contextual environment produces a particular kind of embodied experience by engaging movement as well as the other senses’ (Dovey, Abba, and Pullinger 2021, 13). In light of our audience, we anticipate that some of our users actually may do so within healthcare spaces, while others may use the books to practise close, embodied looking, thereby using that process to think about hospitals using their own remembered experience of such places.

**Figure 4:**
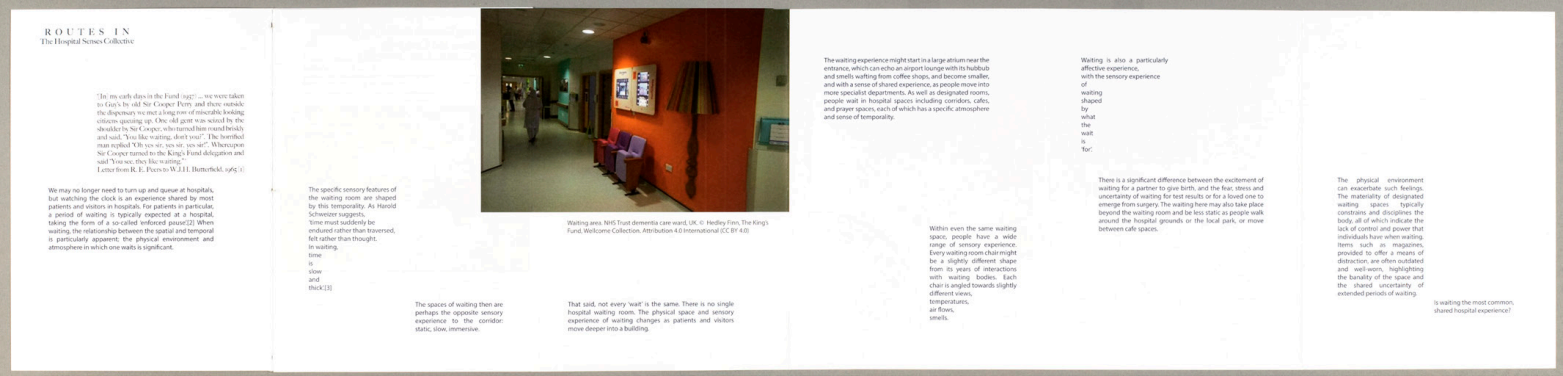
Foldout of waiting page. Full text available to read at: https://hospitalsenses.co.uk/waiting-times-routes-in/. Republished under CC BY-NC.

The booklets thus offer a relation to artists’ books, where users must engage slowly to attend to its uniqueness and fragility. We make this comparison without claiming that our booklets constitute artists’ books. We have an initial print run of 100 and our booklets are not ‘one-off’ pieces of art, though it is worth noting that artists’ books can also come in multiple editions. Each of our booklets is to some extent a unique object within the pack (hand sewn, with handmade additions to some pages), and they bring in many of the sensory modes of enquiry that artists’ books use. Our users know how our booklets are made, and their scarcity is part of their identity and appeal. Indeed, the process of making is key to their eventual form. These booklets required dialogue between making and testing and plenty of risk-taking. It was an iterative process, and they are not *the* final products. We designed and made them over several years, through online workshops and in-person writing retreats. We worked with book designer Tom Abba, who brought expertise in the interactions between books and the world (e.g. Abba et al. 2013 and Dovey, Abba, and Pullinger 2021), and our form evolved in dialogue with the content, and with each other. In places we have included concertina pages, which prompt the user to unfold with care and prompt slow, gradual engagement; the longest of these folds out to four pages, and is used to explore the theme of ‘waiting’ (figure 4).

This use of form has some parallels with works such as *Dependency* (2016) by Pauline Lamont-Fisher, which uses the concertina form to chart the progress of Alzheimer’s disease. With these concertina folds and other parts of the booklets with a ‘long continuous sequence’ or the ‘look of translucent paper’, the user of the booklets may ‘struggle for access to information’ in a productive way (Drucker 2020, 87). Stella Bolaki argues that ‘reading artists’ books translates into an embodied and often meditative practice that requires an active reader’ (Bolaki 2020, 3). The physical layout and multimedia forms engage the user on a multisensory level, inviting them to take part in the experience and to create meaning. The ingrained delicacy of the hand-sewn pieces, with the vulnerability of the tracing paper and inclusions of cloth, encourage a particularly caring tactility: ‘Like patients’, Bolaki argues, ‘artists’ books have to be examined, touched, unveiled, opened but require physical intimacy and care’ (Bolaki 2009, 85).

**Figure 5:**
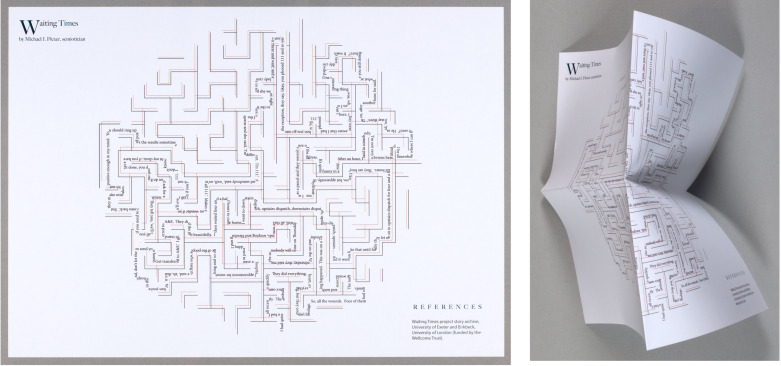
Michael Flexer and Tom Abba, Waiting Times. Republished under CC BY-NC.

The attentiveness to care compels an attentiveness to time. Again, in comparison to artists’ books, they are ‘temporal. We have to spend time with them’ (Drucker 2010 cited in Tuttle and Miller 2020, 54). The set of booklets is just that, a set—one that can be accessed in different orders and at different paces. They are informal; even the pages are not numbered. They were designed as playful objects without formal expectations. Figure 5, for example, is a foldout piece inspired by the non-linear nature of mazes and labyrinths (Higgins 2018). This form offers an intersection between space and time, allowing both to be considered in their full complexity. As discussed above, time in hospitals does not work in ‘snapshots’ nor in a linear way, and it can be hard to capture this in traditional academic forms of narrative that are in themselves linear.

One of the biggest challenges for such multisensory works relates to accessibility. How do we make collaboratively produced, expensively printed, and hand-sewn booklets easily and readily available to those who might want to engage with and use them? One obvious answer is to put them online, free to all to download and keep. But how might we recreate tangibility and touchability online? How do we make the booklets readable, usable, enjoyable for people with specific access requirements? In some ways, there are parts of the booklets that are deliberately difficult to access, and are intended as such to further underline their meaning. Folding out concertinas or reading in different directions are designed to hinder but also help the user make sense or make meaning out of the object. But these forms make it difficult for the booklets to be easily relayed by screen readers for visually impaired people, as just one example. As Egidija Čiricaitė says about photographing artists’ books, ‘they function as a unit of tightly conceptually-bound visual, textual and material elements’ and, as a result, they offer a ‘compelling minefield of issues for anyone trying to document them through photography’ (Čiricaitė 2020, 81). The translation of a ‘three-dimensional haptic artwork’ into two dimensions will always prove imperfect, or at the very least produce a very different thing to the original objects, but we believe this translation is still worth doing. We can also consider such a translation as a further opportunity with which to explore and enhance our understanding.

To address some of these questions, the strategy we employed was to work with an experienced professional Audio Describer, Lonny Evans, to develop a creative response to select readings from the booklets and transform some of the objects’ look and feel into sound. We consider this response or ‘translation’ to be in line with the principles of Universal Design (Steinfeld and Jordana 2012) in which such approaches are not just important for people with specific accessibility needs, but are opportunities with which to improve experiences for all. In her response, which can be accessed on the Hospital Senses website (https://hospitalsenses.co.uk/2019/11/15/hospital-senses-booklets), Lonny uses the audio to capture some of the embodied experiences of interacting with the physical booklets. For example, she reads some sections very slowly to mimic the experience of frustrated reading along folds or through tracing paper. Similarly, as well as audio describing particular images or smells from the booklets which she identified as significant, she has added other creative responses such as non-verbal sounds. This rich audio description is accompanied online alongside photographs of the booklets as objects as well as complete text documents which can be accessed by a screen reader. For those who also have the physical copies, Lonny offers ways to engage with them physically, for example, by inviting people to trace a route through the floor plan printed on the belly band as a way into the booklets. In this way the digital represents a different and complementary experience to handling physical copies rather than being a secondary, lesser element.

Overall, we have produced objects rather than texts. The form is as important as the ideas and the booklets themselves are also creative provocations which encourage a process of imaginative re-engagement with, or remaking of, experience: ‘the participant enters the work’s rhythm, creating a new pattern of relations to themselves and their surroundings’ (Whittaker 2021, 234). While Caroline Levine argues that ‘it is the work of form to make order’ (Levine 2015, 3), our approach has sought instead to disorder, reorder and challenge our forms of knowing and experiencing.

### Encountering the booklets

In 2022–2023 we gathered feedback from users of the booklets, in order to understand how they were encountered and any challenges or limitations to their form. This interaction with users is also a crucial part of any such project, as the making of the booklets and their meaning is an ongoing process that involves crossing boundaries between authors/makers/users. Physical booklets were sent to 10 people from the ‘Senses and Health/care Environments’ network who expressed an interest, from a combination of academia and the field of arts and health. We collected four responses to our survey which give rich qualitative insights into how the booklets are being encountered and used. The feedback indicates that many of the strengths of this kind of physical booklet are also potentially challenging for users. In deliberately making people work to engage with them, for example, the booklets can limit access to the text and its meaning, and the handmade nature of the booklets can restrict their use in potentially valuable contexts such as teaching. The online version of the booklets, and the creative accessible audio description that accompanies them, is therefore an essential counterpart to this kind of project.

The recipients of the booklets were asked to reflect on their encounter with the physical objects, through exploratory questions: Why did you offer to give feedback on the booklets? What was your first impression of the booklets? Talk us through how you engaged with the booklets, in as much detail as you can. Have you engaged with them multiple times? What did you think of the use of different materials and sensory prompts? Do you have any other comments on how they were made? Please give us an example of one element that particularly engaged, interested or surprised you. How did using the booklets make you think or feel about hospital/healthcare environments? Have you used them in your work or practice, or do you envision doing so? How do you think these could be improved? Please share any other thoughts/comments that you have about the booklets.

Responses to these questions indicate that, in many ways, recipients are engaging with the booklets in the ways anticipated above. All respondents noted the aesthetic appeal of the booklets, including their colours and the fact that they felt ‘smooth’, with one additionally noting that the ‘handmade aspect asks for the handler to treat the items with care’. All users noted that they engaged with the booklets slowly, over multiple occasions, with the form of the booklets inviting people to ‘dip in and out’ or ‘skim through’ before engaging with each piece in greater depth and taking time to consider or ‘notice what it sparked’. In terms of the content, everyone appreciated the ‘multi-disciplinary nature’ of the booklets and found the variety stimulating and inviting, and the mixed media and sensory approach added to this appeal. These respondents are already engaged in artistic, sensory, multidisciplinary work—in and out of academia—and this might explain why they were quick to identify features such as ‘multidisciplinary’ pieces and ‘mixed media’. However, the pieces are deliberately not academic or specialist in tone, and we hope that other users would also notice and appreciate the range and variety, such as ‘the mix of creative forms and historical text’ and the impact of the mixed media approach (‘The smell of the laundry one stays with you!**’**).

Respondents brought their own interests and experiences when engaging with the booklets, thereby co-producing their meaning. One commented, for example, that ‘so many of them touch on aspects that emerged within my own research – and they have made me reflect more on this and on my own experiences of care settings – returning as I often do to thoughts of how it is to ‘wait’’. It is significant that each user chose a different example for a piece that ‘particularly engaged, interested, or surprised you’. In that sense, the booklets have achieved their intended aim, of being a collaborative, interactive object with which people can engage in ways that are meaningful to them, whether in relation to their work or their lives. One respondent also noted—in response to the question about a piece that ‘engaged, interested, or surprised you’—that ‘due to the design, I can pick up the booklets on multiple occasions, and … gain something new’. This comment is in part a reflection on the variety of form and content in the booklets, but also indicates that people might engage differently with them over time and find that new pieces draw their attention depending on their own feelings on a given day. The booklets are thus read and engaged with in a different way to traditional academic medical humanities pieces, which often invite being only read once and having a single, clear meaning. The booklets offer, instead, prompts and provocations that are only given meaning in co-production with the user.

There were some critiques, mainly focused on issues surrounding the availability of the booklets. Respondents felt that it would be useful for more people to be able to access the booklets. This is a challenge because their handmade and sensory nature makes them expensive and very time-consuming to construct, and physical versions of the booklets are in short supply. The solution, as outlined above, has been to take photographed versions of the physical booklets and host them online alongside creative audio description, both of which are intended to make them more widely available without losing the physicality and scale of the booklets. While engagement with the design is an important part of the co-creation of meaning, it is important that design enhances rather than *obscures* content. Web pages might provide supporting text for those reading the physical booklets, in terms of providing text in a more ‘straightforward’ format for those who are distracted by the design:

The design of the booklets is both its strength and weakness. The strength of the design aspect is it makes the books enjoyable to move through and investigate. The negative is that the design sometimes takes centre stage above the content of the text. For example, where text has been graphically designed to move around the page, I would read a few lines to get the gist, but rarely the whole excerpt.

Respondents working within academia also noted the potential value for engaging students, a pedagogical approach which might fit with emerging models of creative and ‘embodied inquiry’ (Leigh and Brown 2021). For example, one person noted that ‘I will look forward to using them in teaching and getting students to also think about the small elements/details of care and how important these are – and how important the senses are’. The engaging nature of such booklets thus makes them a valuable tool for provoking conversation and thought in a range of contexts, offering intellectual stimulation in an accessible form; they can support and complement more traditional academic outputs. Again, respondents noted some of the potential downsides of their design here, for example, whether the ‘fragile’ hand stitching would tolerate being handled by multiple students. While the handmade nature of the booklets invites care, it also has inbuilt limitations: would the invitation to take care of these booklets make people hesitant to share them widely?

Overall, the feedback on the booklets has been extremely positive. There were some suggestions for tweaks to individual contributions in addition to the more general comments made above, but all respondents emphasised that they were ‘engaging and enriching’, ‘wonderful’, ‘beautiful’ and ‘I loved them as they were’. This feedback supports this article’s argument that there is great value in going beyond traditional academic forms. Academics and arts/health practitioners alike, in our small feedback group, found great value in the booklets. Their form invited a new kind of thinking, interaction and co-production, which respondents could envisage being useful in contexts such as teaching and healthcare.

It is also, of course, important to recognise the limitations of any project. Our feedback group came with an existing interest in the booklets’ subject matter and an open mind to creative formats and ways of thinking, and it would not be right to draw conclusions about the booklets’ wider value or appeal based on these responses alone. The challenges of availability, accessibility and durability were also quite rightly recurrent themes in feedback. The online versions of the booklets address these issues, but they do limit many of the tactile and sensory aspects, and cannot be viewed as a direct or equivalent substitute to the booklets. Ideally, the online versions will be used as a complement to the physical booklets rather than a substitute for them, but time and expense does make the mass production of such publications challenging. Projects like this, then, will always involve some compromises between reach, accessibility and design.

## Conclusions

In 2011, Angela Woods offered a ‘provocation’ to medical humanities scholarship on the ‘limits of narrative’. In this piece, she noted that ‘narrative […] enjoy[s] an exceptionally privileged role in medical humanities scholarship’ but that there are limits to the value of thinking only in narrative terms (Woods 2011, 73). Woods opened up an important space for debate and a valuable critique of the predominance of ‘narrative’ in medical humanities research, yet academic work in the medical humanities remains largely narrative in format unless framed as ‘public engagement’ or ‘outreach’. Given our focus on the multisensory nature of healthcare spaces, we wanted to find a way to explore and create along more-than-narrative lines, and provide opportunities to creatively encounter spatial, sensory and emotional ideas. We hope that these booklets will be a catalyst for further provocations and reflections.

We recognise that there are numerous barriers to the production of work of this kind, not least finance. The booklets were an expensive and time-consuming process, which relied on collaboration with skilled experts and practitioners. For instance, one of our coauthors worked closely with Tom Abba on the design and format of the physical booklets. This close working was possible due to their creative practice and expertise using design software. Working in this way created room for continued collaboration and nuanced dialogue that would be challenging in teams without such skill sets. Similar creations would need not only significant funds, but also understanding for the level of experimentation, trial and error, and risk-taking which creative pieces often require. There needs to be sufficient investment in interdisciplinary teams to produce the work fairly and effectively, but also investment in long-term collaboration. Successful collaboration, especially when multidisciplinary, and bridging inside and outside the academy (not to mention the barriers to research within clinical contexts) only succeed when working relationships are able to flourish over time. Furthermore, such a project should not be an add-on to a larger, more traditionally academic project, but instead built in from the beginning. It was due to a range of factors, and the accommodations and flexibility of our funding, which meant we were able to deliver this project. Such funding flexibility is rapidly disappearing, so will not be a route for others.

The collaborative process that made producing these booklets possible, then, requires financial and institutional support of a kind that is difficult to find in the contemporary academy. This is true in spite of universities’ and funders’ vociferous, explicit commitment to interdisciplinary and collaboration, and to things like public engagement, research impact, and knowledge exchange. As we have argued in this article, the booklets were designed as a series of prompts or provocations, created to engender users’ responses, considerations and reflections. But as we hope we have made clear, the booklets were not just fruitful objects for those who received them once they were ‘complete’, but profoundly generative things to make and create. Their value is not just in the end product (for want of a better word), but in the *process*—even if that process is difficult, if not impossible, to be measured or assessed according to the metrics currently available to academics. This project is not, therefore, an exercise to present or disseminate research, but an effort to *do* research differently.

## Data Availability

Data may be obtained from a third party and are not publicly available. Most underlying data are available from sources referenced throughout this paper. Underlying data from surveys are quoted in the paper, but participants did not consent to the publication of full survey responses.
